# Development of Low-Cost Wireless Sensing System for Smart Ultra-High Performance Concrete

**DOI:** 10.3390/s21196386

**Published:** 2021-09-24

**Authors:** Huy-Viet Le, Tae-Uk Kim, Suleman Khan, Jun-Young Park, Jong-Woong Park, Seung-Eock Kim, Yun Jang, Dong-Joo Kim

**Affiliations:** 1 Department of Civil and Environmental Engineering, Sejong University, 98 Gunja-dong, Gwangjin-gu, Seoul 143747, Korea; lehuyviet.mdc@gmail.com (H.-V.L.); rlaxodrn1117@naver.com (T.-U.K.); sekim@sejong.ac.kr (S.-E.K.); 2 Department of Civil Engineering, Hanoi University of Mining and Geology, Hanoi 100000, Vietnam; 3 Department of Smart Cities, Chung-Ang University, Seoul 06974, Korea; sulemank137@cau.ac.kr; 4 Department of Civil and Environmental Engineering, Chung-Ang University, Seoul 06974, Korea; pjy5451@gmail.com; 5 Department of Computer Engineering and Convergence Engineering for Intelligent Drone, Sejong University, 98 Gunja-dong, Gwangjin-gu, Seoul 143747, Korea; jangy@sejong.ac.kr

**Keywords:** wireless sensing system, smart ultra-high performance concrete, self-stress sensing, self-damage sensing, damage crack sensor, structural health monitoring

## Abstract

This study proposes the development of a wireless sensor system integrated with smart ultra-high performance concrete (UHPC) for sensing and transmitting changes in stress and damage occurrence in real-time. The smart UHPC, which has the self-sensing ability, comprises steel fibers, fine steel slag aggregates (FSSAs), and multiwall carbon nanotubes (MWCNTs) as functional fillers. The proposed wireless sensing system used a low-cost microcontroller unit (MCU) and two-probe resistance sensing circuit to capture change in electrical resistance of self-sensing UHPC due to external stress. For wireless transmission, the developed wireless sensing system used Bluetooth low energy (BLE) beacon for low-power and multi-channel data transmission. For experimental validation of the proposed smart UHPC, two types of specimens for tensile and compression tests were fabricated. In the laboratory test, using a universal testing machine, the change in electrical resistivity was measured and compared with a reference DC resistance meter. The proposed wireless sensing system showed decreased electrical resistance under compressive and tensile load. The fractional change in resistivity (FCR) was monitored at 39.2% under the maximum compressive stress and 12.35% per crack under the maximum compressive stress tension. The electrical resistance changes in both compression and tension showed similar behavior, measured by a DC meter and validated the developed integration of wireless sensing system and smart UHPC.

## 1. Introduction

The use of smart construction materials (SCMs) with self-stress and damage sensing abilities in buildings and civil infrastructure for structural health monitoring is promising. SCMs are expected to overcome many limitations, including low durability, high cost, and localized sensing ability of current monitoring systems utilizing commercially attached or embedded sensors with wired connections to data acquisition systems [1]. The self-sensing abilities of SCMs are based on changes in their electrical resistance [2]. Cracks are generated under external loads, as the strain in SCMs changes. The electrical resistance or resistivity of SCMs changes owing to variations in the electrically conductive networks of functional fillers (FFs) within the SCMs [1,2]. Furthermore, various FFs have been added to SCMs to increase the conductive networks in SCMs under external loads [3,4,5,6,7].

Lee et al. [3] recently developed smart ultra-high performance concrete (S-UHPC) with a high self-stress sensing capacity of up to 60 MPa compressive stress by utilizing fine steel slag aggregates (FSSAs). The S-UHPC could monitor the loss in prestressing force in prestressing steel (PS) tendons when applied to the anchorage zone of the tendons [4]. Furthermore, Le et al. [5] reported that S-UHPCs with steel fibers helped successfully detect the location of cracks or damage within tensile specimens of S-UHPCs under direct tension by measuring the corresponding electrical resistivity. However, the practical application of SCMs, including S-UHPCs, to buildings and infrastructures under external loads is still challenging [6]. Additionally, to apply S-UHPCs for reinforced concrete structures with steel reinforcements, the effect of the combination between functional fillers and steel reinforcements on sensing characteristics should be further investigated, because steel reinforcements also have highly electrical conductivity.

The electrical resistance or impedance of SCMs has generally been measured using direct current (DC) or alternative current measurement methods that require relatively expensive devices. Additionally, the experimental results reported in previous studies were mainly obtained from single-channel measurements. This limits the practical application of SCMs to buildings and infrastructure for structural-health monitoring systems that require multiple sensing positions. Recently, Downey et al. [7] reported multiple sensing systems using biphasic DC measurements. Furthermore, Le et al. [5] performed self-damage sensing at multiple positions using multiple DC measurements.

In order to address the practical issue of sensing in the SCMs, wireless sensing could be a solution attributed to its inexpensive sensing system and simplicity in instrumentation for distributed sensing for electrical resistance measurement of SCMs. Wireless sensing systems (WSSs) have been applied to the field of structural health monitoring [8,9,10,11] and proven their excellence in sensing and embedded data processing compared to traditional wire-based counterparts [12,13,14]. The embedded computing power in the wireless sensor system allows real-time data acquisition and processing of measured data transmitted through wireless communication. Owing to distinct embedded processing and transmission ability, wireless sensors could be used for long-term monitoring of SCMs when these are deployed in the field. Studies on the development of WSSs integrated with SCMs have been conducted to overcome the limitations related to wired data acquisition systems. Lynch et al. [15] and Hou et al. [16] developed a WSS for measuring the electrical responses of engineered cementitious composites (ECCs) containing a low volume fraction of short fibers (polymer, steel, and carbon) and reported the self-sensing response of ECC under tension test. In 2010, Han et al. [17] developed a Zigbee-based WSS to acquire the electrical signals of an SCM containing nickel powder under a tension load. Lee et al. [18] developed a WSS module composed of LoRa(long-range) communication protocol and electrical resistance sensing system for cement composites incorporating 1.0 vol% multi-walled carbon nanotubes (MWCNTs) as a conductive filler. The performance of the developed wireless module was validated through a compression test.

This study aimed to develop a low-cost WSS composed of a simplified resistance sensing circuit for electrical resistance measurement of S-UHPC, a power management circuit for long-term operation, and Bluetooth low energy (BLE) for real-time data transmission. The S-UHPC was fabricated using Steel fibers, fine steel slag aggregates (FSSAs), and multiwall carbon nanotubes (MWCNTs) together as FFs to develop the self-sensing ability. A lab-scale experiment was conducted to validate the performance of the WSS integrated with S-UHPC in detecting stress changes and damage under compression and tension. The results were compared with the wired DC measurement. The specific objectives of the study are as follows: (1) to develop a low-cost sensing circuit for measuring the electrical resistance of S-UHPC; (2) to investigate the change in the electrical resistance of S-UHPCs under load using the developed WSSs; and (3) to validate the self-sensing capacity of S-UHPCs using the proposed WSSs.

## 2. Proposed Wireless Sensing System for Multiple Electrical Resistance Measurements

This section introduces a low-cost WSS deploying a BLE beacon integrated microcontroller unit (MCU), an electrical resistance sensing circuit, and a power management circuit. Additionally, a BLE packet for data transmission is proposed to allow real-time sensing using the proposed WSS.

### 2.1. Microcontroller Unit

Adafruit nRF52840 ItsyBitsy (Adafruit Industries, New York, NY, USA) was selected as the MCU to prototype the proposed low-cost WSS. The nRF52840 ItsyBitsy has a processor chip (nRF52840) developed by Nordic Semiconductor with a 32-bit ARM^®^ Cortex™-M4 core, and a clock speed of 64 MHz enables fast data acquisition and real-time processing. Another feature of nRF52840 ItsyBitsy is the built-in wireless connectivity support using Bluetooth 5 technology for wireless communication. This allows long-range data transmission with a high bit rate (2 Mbps) and low power consumption (14 mA).

### 2.2. Electrical Resistance Sensing Circuit

**Figure 1:**
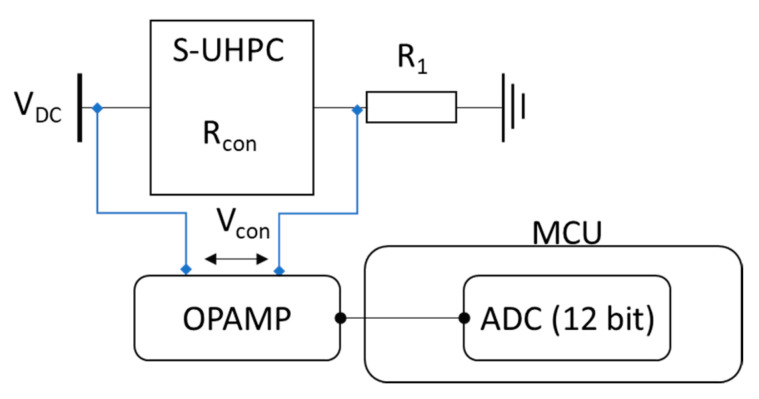
Block diagram of the resistance sensing circuit.

A sensing circuit for measuring the resistance was developed using a two-probe method. However, the electrical resistance measured via the two-probe method is potentially higher than the actual electrical resistance value of the composites due to the contact resistance effect. The changes in the resistance of the composites under external loads are not affected by the two-probe method [18,19]. In addition, due to the extremely high electrical resistance of the S-UHPC (i.e., 1 MΩ) used for integration, the voltage output from the S-UHPC was out of the internal 12-bit ADC range. Therefore, the electrical resistance measurements in this study were performed using the two-probe method. Figure 1 shows a block diagram of the resistance sensing circuit using the two-probe method. The operational amplifier (Op-Amp) is used in the non-inverting buffer configuration to stabilize the voltage *V_con_*. The Op-amp provides high input and low output impedance to stabilize the output voltage results in stable converted resistance.

The circuit includes a voltage divider circuit (*V_con_*) that consists of a battery supply (*V_DC_*), balancing resistance (*R*_1_), and electrical resistance of the S-UHPC specimen (*R_con_*). Hence, *V_DC_* can be used to calculate *R_con_* using known *V_DC_*, *R*_1_, and *V_con_*. In particular, *V_con_* is the measured voltage across the S-UHPC by the internal 12-bit ADC as follows:

(1) $$R_{con}=\frac{V_{con}\times R_{1}}{V_{DC}-V_{con}}$$

Embedded software is developed to obtain *R_con_* directly using Equation (1). The nRF2840 ItsyBitsy starts data acquisition of *V_con_* at 1 Hz using a built-in 12-bit ADC, and the resistance value of *R_con_* is calculated based on Equation (1). The resistance value of *R*_1_ is 1 MΩ that is similar to S-UHPC for accurate resistance measurement.

### 2.3. Power Management Circuit

**Figure 2:**
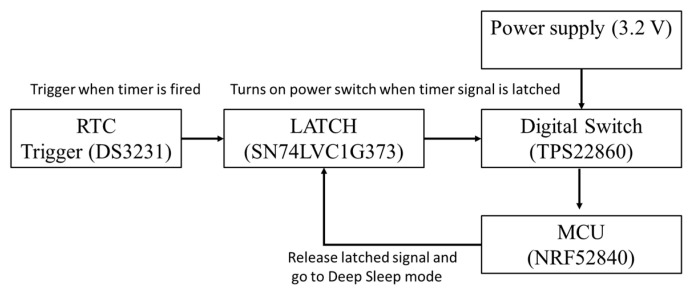
Functional block diagram of power management circuit.

A power management circuit is designed to enable long-term monitoring by minimizing power consumption and increasing stability. The power efficiency can be significantly improved by introducing Deep Sleep, where power is removed from most chips, except for the power management circuit. The power management circuit is triggered by a real-time clock (RTC) that turns on the digital power switch and latches the power for MCU. The latch is controlled by a GPIO signal controlled by the MCU and latched at the startup of the MCU. After processing in the MCU is completed, MCU releases the latch to go into Deep Sleep mode (Figure 2).

Since RTC is an active low device the generated signal on trigger was then inverted by NOT-gate and connected to the latch (SN74LVC1G373 from Texas Instruments, Dallas, TX, USA). The digital switch (TPS22860 from Texas Instruments, Dallas, TX, USA) controlled by the latch signal is to provide the regulated voltage from MCP1725 by microchip delivered to MCU. All the devices considered in the design of the power management circuit have low power consumption. Overall power consumption during sleep mode by each component is listed in Table 1. Note that the electrical resistance sensing circuit is supplied with constant power to avoid polarization time in S-UHPC. The power consumption in the electrical resistant sensing circuit was estimated at 1.6 μA (i.e., supplied power of 3.2 V/total resistance by the S-UHPC and R1). The total power consumption in the Deep Sleep mode is 242.6 μA and in the activation mode is 10.2 mA. Given a 10,000 mAh battery and ten sensing events with 60 s of operation, the developed WSS can last 3.68 years, consuming an average of 310 μA.

**Figure 3:**
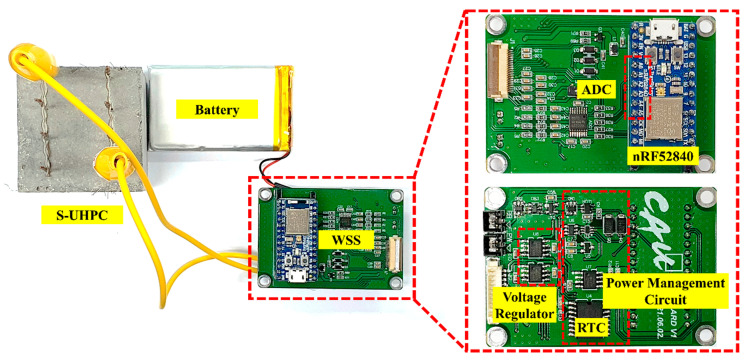
Developed wireless sensing system.

When Deep Sleep mode is disabled, the WSS is designed to calculate *R_con_* and broadcast its data through the BLE beacon. In the power-saving mode, the WSS runs sensing, data processing, and data transmission for one cycle and then goes into Deep Sleep mode until the RTC timer is activated. The developed WSS is shown in Figure 3.

### 2.4. Bluetooth Communication

**Figure 4:**
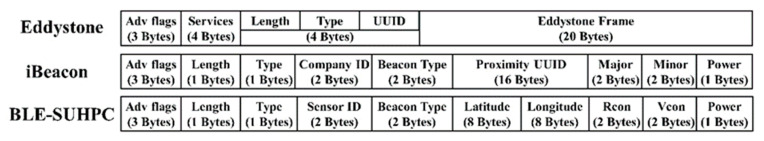
Packets used in different protocols for BLE communication.

Bluetooth Low Energy (BLE) is a form of wireless communication designed especially for short-range communication. It allows communication between BLE-compatible devices such as smartphones, smartwatches, and laptops. A BLE beacon is a device that uses BLE advertising mode to broadcast data packets, including a universally unique identifier selected by a BLE-compatible device to connect and receive data packets with low power. The communication requires two devices: a peripheral node that sends data and a central node that receives data and parses content to extract information. The advertising mode is unique, given that the connection protocol does not require pairing with the central node for data exchange. It can broadcast specified information without a direct connection to a central node. The mode uses a generic access profile (GAP) layer to broadcast data in the form of packets. The BLE-enabled peripheral node uses a specified packet format for data transmission, which was designed by considering special interest group specifications. Eddystone and iBeacon [20] correspond to two reference protocols that are commonly followed in designing data packets used for BLE communication, and both protocols exhibit the same packet size of 31 bytes (see Figure 4). The first protocol for this type of communication corresponded to iBeacon, which Apple developed in 2013 to broadcast data for neighboring devices. Eddystone was introduced two years later by Google.

This paper proposes a BLE-SUHPC protocol that effectively broadcasts packets comprising information related to the process at the WSS (i.e., resistance, voltage, location of the device, and transmission power) through the BLE advertisement mode. The packet in the proposed protocol is modified from iBeacon, given its ease of use. The Company ID, proximity UUID, and major and minor in iBeacon were modified to send sensor ID, GPS (latitude and longitude) of BLE-SUHPC, *R_con_*, and *V_con_*, respectively. The definition of the BLE-SUHPC packet is as follows.

- Sensor ID (2-Bytes): Identification of the peripheral nodes S-UHPC (e.g., BLE-SUHPC).
- GPS coordinate: (16-Bytes): GPS (latitude (8-Byte) and longitude (8-Byte)) of the BLE-SUHPC.
- *R_con_* (2-Bytes): Resistance value of the S-UHPC.
- *V_con_* (2-Bytes): Voltage measured across the S-UHPC.
- Power (1-Byte): Transmission power, signal transmission strength of communication.

**Figure 5:**
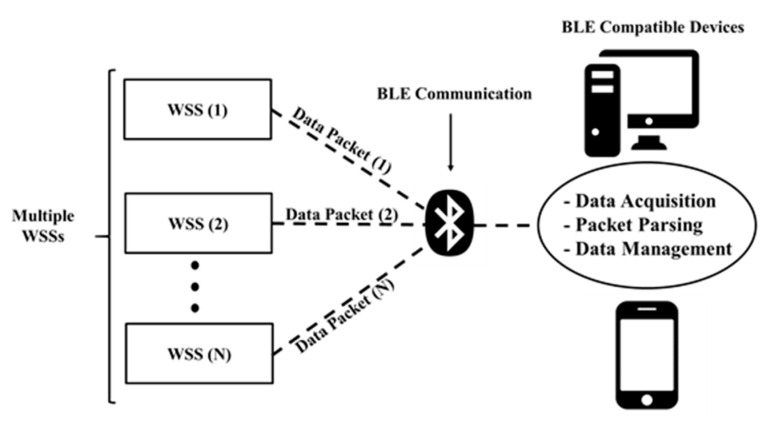
WSS with multiple channel measurements.

The data transmission rate was set to one packet per second. The BLE advertising mode was set as advertised, and two BLE-SUHPCs were developed for multichannel measurement, each distinguished through unique sensor ID and GPS coordinates. The communication protocol allows the connection of multiple peripheral nodes to a single central node. Figure 5 illustrates the devices, along with their functions, involved in the monitoring of S-UHPC. The central node (receiver) distinguishes BLE-SUHPC by its unique sensor ID and extracts the *R*_con_ value by checking redundant packets to minimize packet loss.

## 3. Smart Ultra-High-Performance Concrete Specimen

Smart ultra-high performance concrete (S-UHPC) has been recently developed and validated for its high-sensitive sensing capability [3,21,22,23,24]. The S-UHPC contained fine steel slag aggregates (FSSAs) instead of silica sands or replacing the 50% of silica sands in normal UHPC composition in order to uniformly distribute electrically conductive functional fillers within the matrix. In addition to FSSAs, the S-UHPC also contained discrete steel fibers to maintain the linear elastic behavior until high compressive strength (60 MPa). The S-UHPC has demonstrated the self-damage sensing capacity under tension based on the multiple micro-cracking response [25]. In the study, two types of matrices (M1 and M2) were used to manufacture the S-UHPCs for integration with the WSS. Matrix performance with different sensitivity was compared and is provided in Table 2.

**Figure 6:**
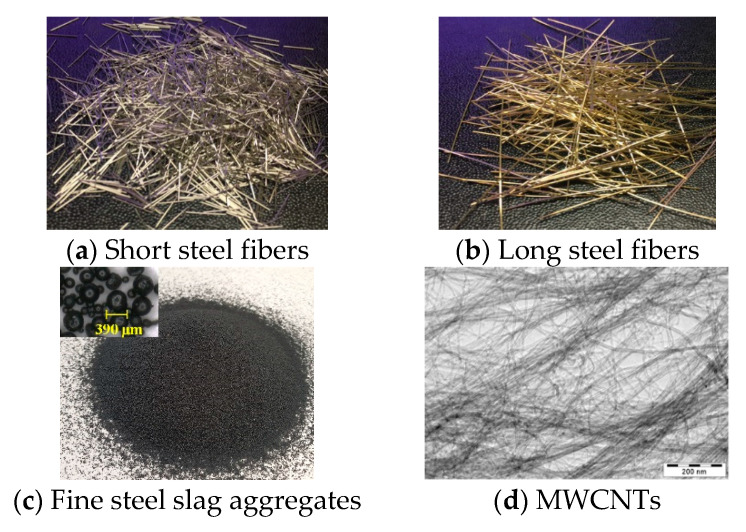
Images of the functional fillers.

M1 contained FSSAs and steel fibers, while additionally, M2 did CNTs. M1 produced slightly higher compressive strength, whereas M2 showed higher conductivity owing to a better conductive network. The proposed WSS measured the electrical response of both M1 and M2 and the reference DC measurement method using a digital multimeter (Fluke 8846A) to validate the self-sensing capacity of the S-UHPC. Table 3 lists the properties of the FFs. The matrix compositions of S-UHPCs containing steel fibers and FSSAs without MWCNTs (M1S and M1L for S-UHPCs with short and long fibers, respectively) were adopted from [21] to obtain high durability and self-sensing capacity of the S-UHPC. The compositions of M2S and M2L included M1S and M1L incorporated with MWCNTs (0.1% weight of cement), respectively. The purity of the MWCNTs (length: 0.01 mm and diameter: 10 nm) corresponded to >99 wt.% with a specific surface area in the range 150–200 m^2^/g. The silica sand had an average diameter of 0.2 mm. Spherical FSSAs (maximum diameter: 0.39 mm) was used instead of silica sand in the conventional UHPC matrix. The FSSAs to cement ratio (by weight) corresponded to 0.5. The FSSAs comprised an electric arc furnace steel slag using slag atomizing technology produced by Ecomaister Co. Ltd. A polycarboxylate-based superplasticizer with 30% solids and 70% water was used to improve the workability of the UHPC mixtures. Figure 6 shows images of the steel fibers, FSSAs, and MWCNTs.

**Figure 7:**
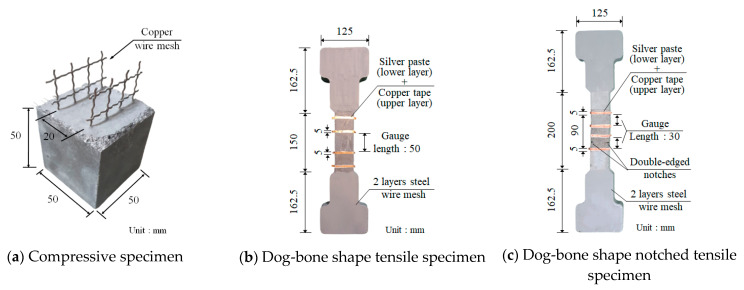
Designs of the sensing specimens.

To investigate the self-sensing capacity of the proposed S-UHPCs, three types of tests were carried out as shown in Figure 7: cubic specimen for compressive tests (50 × 50 × 50 mm^3^) in Figure 7a; dog-bone shape specimen for tensile tests in Figure 7b; the notched specimen for tensile tests in Figure 7c. Two copper wire mesh (height: 70 mm, width: 45 mm, diameter: 1 mm, and mesh interval: 10 mm) were embedded into the cube compressive specimens as electrodes with a 20 mm distance. In the tensile and notched tensile specimens in Figure 7b,c, silver paste and copper tape were attached on the surfaces as electrodes with a 50 mm distance.

For the multi-channel wireless sensing test, double-edged notches (depth: 8 mm) were prepared for the tensile test. One sensor was attached to the area without damage, while the other was installed on the area with a double-edge notch. Silver paste and copper tape, used as electrodes, were attached to four surfaces with a 30 mm distance of each electrode. Moreover, two layers of steel wire mesh were reinforced at each end of the tensile specimen to prevent failure beyond the gauge length, as shown in Figure 7b,c.

The S-UHPC specimens were prepared using a Hobart-type laboratory mixer (20l capability). Silica sand, silica fume, cement, silica powder, and FSSAs were initially dry-mixed for 5 min in preparing the S-UHPC mixtures without MWCNTs. Then, water was gradually added to the mixture and mixed for another 3 min. A superplasticizer was slowly added, which was then mixed for another 3 min. When the flow value of the mixture was in the range 240–260 mm (Table 2), the mixture exhibited suitable workability for uniform fiber distribution, and the fibers were manually dispersed into the mixture and further mixed for 1 min. However, for the S-UHPC specimens containing MWCNTs, the MWCNTs were initially distributed in water using a sonicator with an amplitude of 50% for one hour. The solution containing MWCNTs was mixed with half of the superplasticizer using a stirring bar. The cement, silica sand, FSSAs, silica fume, and silica powder were dry-mixed for 5 min before adding the solution of MWCNTs. The solution of MWCNTs and superplasticizer was gradually added to the dry mixture and mixed for another 3 min. The remaining half of the superplasticizer was then added and mixed until the mixture exhibited suitable workability. The fibers were subsequently added to the mixture and mixed for 1 min.

After mixing, the S-UHPC mixtures with short fibers (6 mm in length) were poured into cubic molds (50 × 50 × 50 mm^3^) for the compressive specimens. To prepare tensile specimens, as the length of fibers is 30 mm while the cross-section area of specimens is 25 × 50 mm^2^, the effect of fiber distribution should be considered. S-UHPC mixtures with steel fibers were poured from one end to other end of dog-bone-shaped molds with two layers during casting. Thus, fibers would be dispersed according to applied load direction, i.e., the gauge length direction. The dispersion of fibers in tensile specimens was assumed as 2D distribution. A slight vibration was applied using a vibration table to reduce the air bubbles within the specimens. Two copper wire meshes were embedded into the compressive specimens. Specimens without embedded copper wire mesh were also prepared to test the compressive strength of the S-UHPCs (Table 2). All specimens were covered with a plastic sheet in the laboratory at 20 ± 2 °C for 48 h before demolding. The de-molded specimens were then cured in hot water (90 °C) for 3 d.

## 4. Experimental Validation

**Figure 8:**
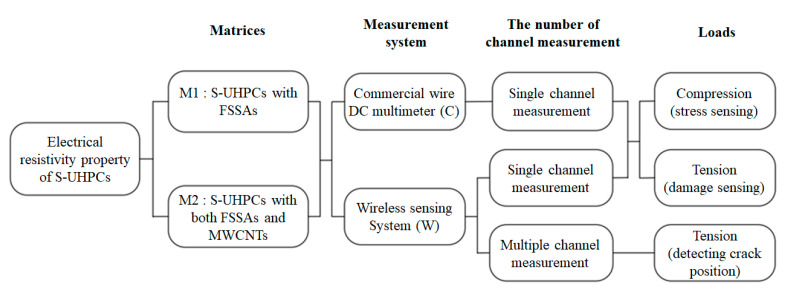
Experimental program.

**Figure 9:**
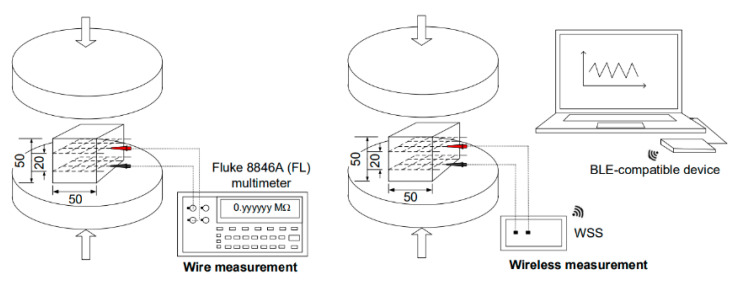
Electrical resistance measurement using wire and WSS measurement under compression.

Figure 8 shows an overview of the experimental program designed to investigate the feasibility of the developed WSS integrated with S-UHPCs. Both S-UHPCs with and without MWCNTs were used to investigate the effects of adding nano-FFs on the self-stress and damage sensing ability of S-UHPCs under external loads. Short, smooth steel (S) fibers (length: 6 mm and diameter: 0.2 mm) were used as compressive specimens [3,21]. Conversely, long smooth steel (L) fibers (length: 30 mm and diameter: 0.3 mm) were used as tensile specimens to generate tensile strain hardening accompanied by multiple microcracks [26]. A digital multimeter (Fluke 8846A) with a wired connection was used to compare the results with WSS results (Figure 9).

### 4.1. Test Setup

**Figure 10:**
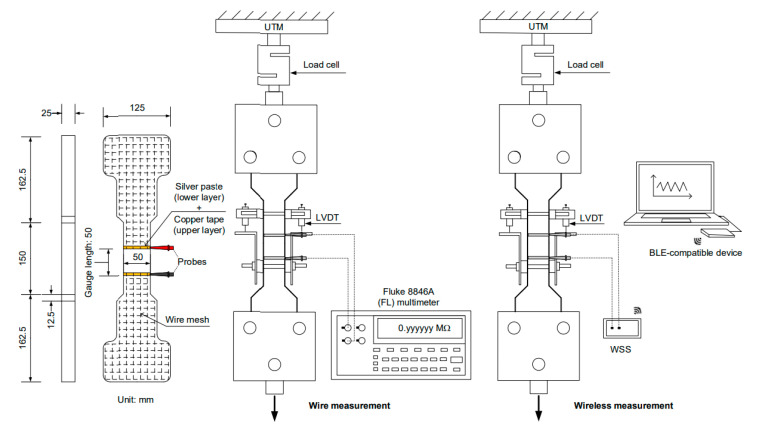
Electrical resistance measurement using wire and WSS measurements under tension.

**Figure 11:**
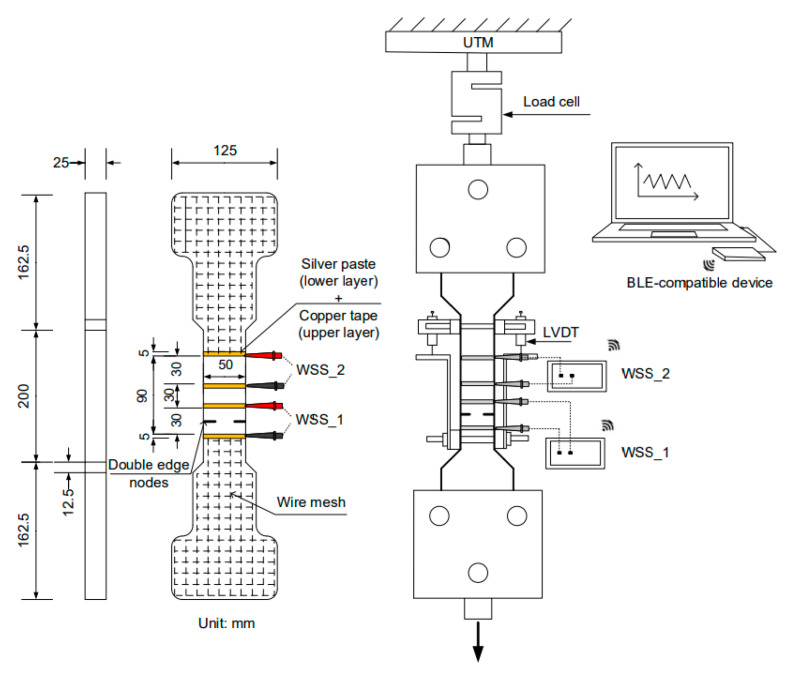
Test setup for multiple electrical resistance measurements using WSS under tension.

Figure 9 shows the setup for single-channel electrical resistance measurements using WSS and DC measurements under compression, while Figure 10 shows the setup under tension. Figure 11 shows the multiple-channel electrical resistance measurements using WSS under tension. A universal testing machine (UTM, 300-tons capacity) was used to apply a compressive or direct tensile load to the specimens by maintaining a displacement speed corresponding to 1 mm/min during loading. The load was measured using the embedded load cell in the UTM for compressive specimens and an attached load cell with 49,050 N (5000 kgf) capacity for tensile specimens. The elongation of the tensile specimens under tension was measured using LVDTs attached to the gauge length of the specimens (Figure 10 and Figure 11).

The electrical resistance of the specimens under load was measured using the developed WSS and DC measurements with two -probe method. Prior to the tests, the electrical resistances of all specimens were stabilized for 50 min to minimize the polarization effect [27]. A WSS with a single-channel measurement system was used to measure the electrical resistance response of S-UHPCs (compressive specimens in Figure 9 and tensile specimens in Figure 10). Subsequently, a WSS with two-channel measurements was used to measure the electrical resistance of the cracked and crack-free parts of the specimens, as shown in Figure 11. The first WSS (WSS 1) was used for the gauge length with a double-edged notch, while the second WSS (WSS 2) was used for the gauge length without a double-edged notch. In the WSS, the measured electrical resistance data of S-UHPCs were transferred to a laptop via Bluetooth Beacon.

The electrical resistivity was calculated using Equation (2), as follows: The electrical resistance (*R*) was affected by the distance between the electrodes (*L*, cm) and cross-sectional area (*A*, cm^2^). Conversely, the electrical resistivity (*ρ*, kΩ·cm) corresponds to material property.

(2) $$\rho =R\cdot \frac{A}{L}$$

### 4.2. Results and Discussion

#### 4.2.1. Polarization of S-UHPCs and Sensing Ability of WSS

Before the polarization test, the electrical resistance value measured by the WSS and the DC meter were compared by connecting the same resistance of 1000 kΩ. For 5 min of measurement, the average resistance measured from the WSS was 1002.5 kΩ, while the DC meter’s value was 998.3 kΩ showing a highly accurate result with 0.42% error. 

**Figure 12:**
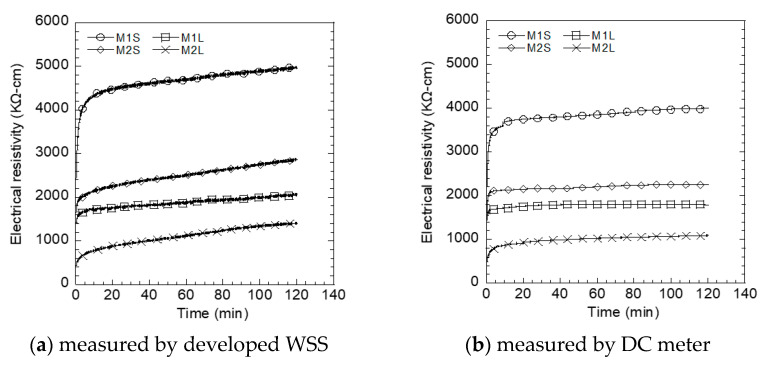
Polarization of S-UHPCs.

Figure 12 shows the electrical resistivity polarization of S-UHPC using a single BLE-SUHPC measurement (Figure 12a) and a DC meter (Figure 12b) in a controlled condition of a chamber (20 °C temperature and 80% humidity). The polarization time of the specimens was approximately in the range 20–40 min for all measurements, irrespective of the DC or BLE measurements. Le et al. [5] also reported a polarization time corresponding to 30 min for S-UHPCs containing short steel fibers. In addition, Nguyen et al. [26] reported that the polarization time of steel fiber-reinforced HPC was approximately in the range 20–30 min.

**Figure 13:**
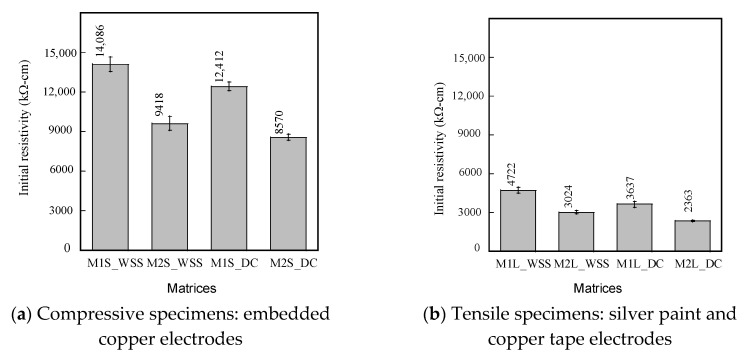
Initial electrical resistivity of S-UHPCs.

Figure 13 compares the initial electrical resistivity of different S-UHPCs with different fiber lengths and electrode types measured using the WSS and DC multimeter in a laboratory condition prior to applying external loads. In general, the electrical resistivities of S-UHPCs with MWCNTs (M2S or M2L) were lower than those of S-UHPCs without MWCNTs (M1S or M1L) irrespective of the measurement method, including the WSS and DC multimeter. The lower electrical resistivities of the S-UHPCs with MWCNTs were attributed to the addition of highly electrically conductive MWCNTs. In S-UHPC matrices, the FSSAs (particle type conductive material) produced tunneling conduction as well as contact conduction, while steel fibers (fiber type conductive material) mainly did contact conduction between FFs [3,21]. The addition of MWCNTs enhanced the conductive network of the S-UHPCs at the nano level. Consequently, MWCNTs increased tunneling conduction and contact conduction between the FFs in the matrix (steel fibers, FSSAs, and MWCNTs). Furthermore, as shown in Figure 12, the electrical resistivities of specimens with long steel fibers (M1L or M2L) were lower than that of specimens with short steel fibers (M1S or M2S), irrespective of the measurement methods, including the WSS and DC multimeter. Long fibers (length: 30 mm) can easily connect FFs (FSSAs and MWCNTs) and generate a shorter distance between the conductive pathway of FFs in the matrix, consequently resulting in higher electrical conductivity. However, the electrical resistivities of the S-UHPCs from WSS were observed to be higher than those using the DC meter. The electrical resistivity of M1S measured by WSS was clearly higher than that of M1S measured by DC meter, as can be seen in Figure 12. The different electrical resistivity would be due to difference in hardware (e.g., applied current) and software systems of WSS and DC meter. The source of different electrical resistivity measured by WSS and DC meter should be further investigated in future research.

Additionally, the electrical resistance of S-UHPCs was clearly influenced by changing environmental conditions, including temperature and humidity [22]: the electrical resistivity of S-UHPCs significantly decreased (approximately 2.27% per °C) as the temperature increased from 0 to 40 °C, whereas it slightly changed as the humidity increased from 20% to 80% owing to high density in microstructure of S-UHPC matrix. To evaluate the self-sensing characteristics of S-UHPCs at long-term conditions, a further study should be investigated to separate the effect of environmental and external loads based on the big data of environmental effects.

#### 4.2.2. Self-Stress Sensing of S-UHPCs Using WSS and DC Measurements under Compression

The WSS measurement demonstrated the feasibility of measuring the electrical resistivity response under compression. The WSS measurement produced a similar trend in the electrical resistivity response of the S-UHPCs with the DC measurement. When the compressive stress increased, the electrical resistivity of the S-UHPCs significantly decreased. Moreover, the addition of MWCNTs significantly enhanced the self-stress ability of the S-UHPCs.

**Figure 14:**
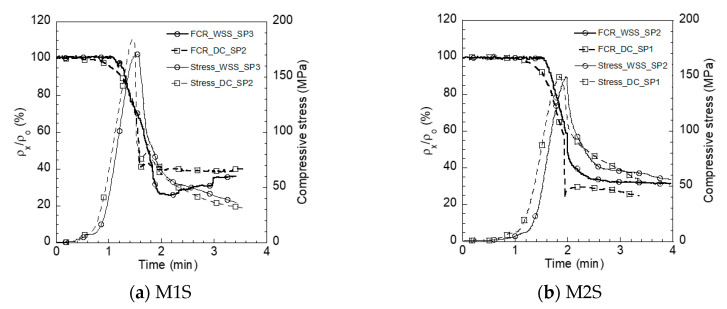
Electrical resistivity response of S-UHPCs under compression.

Figure 14 shows the electrical resistivity responses of S-UHPCs measured via WSS and DC measurements under compression. In general, both measurement systems produced electrical resistivity responses of S-UHPCs that were similar to the response in [3,21]. The electrical resistivity decreased when the compressive stress increased, until the peak stress was reached. The WSS measurements were shown to collect electrical resistivity data in real-time without delay during the test, as shown in Figure 14.

To compare the self-stress sensing capacities of S-UHPCs, the fractional change in resistivity (*FCR*) was calculated at the peak stress position using Equation (3) as follows:

(3) $$FCR_{}=\left|\frac{\rho -\rho_{0}}{\rho_{0}}\right|\times 100=\left|\frac{\Delta \rho_{}}{\rho_{0}}\right|\times 100$$

where *ρ* denotes the electrical resistivity at the determined stress, and Δ*ρ* denotes the change in the electrical resistivity until the determined stress.

**Figure 15:**
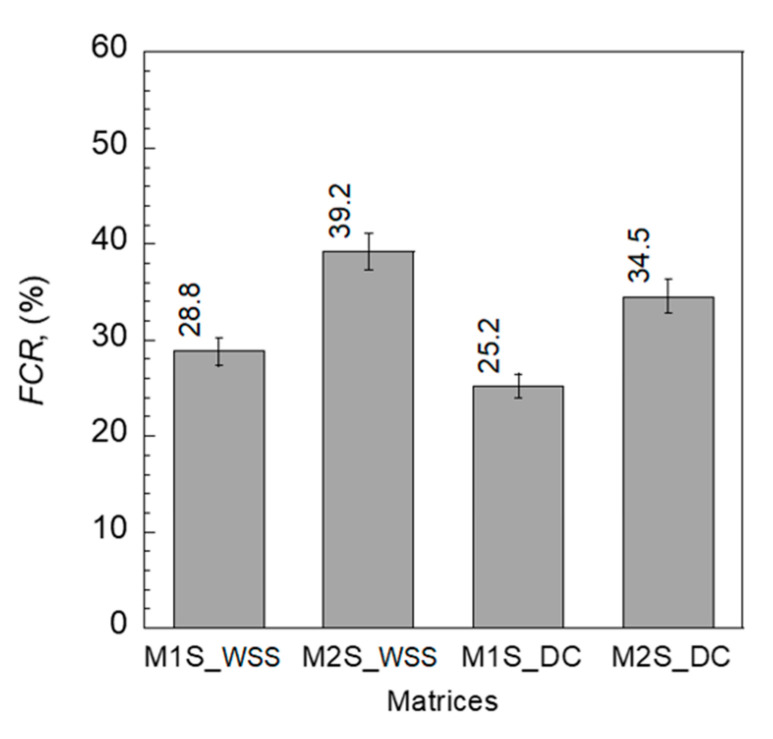
*FCR* at peak stress of S-UHPCs under compression.

Table 4 summarizes Δ*ρ* for the *FCR* of the S-UHPCs at peak stress. The addition of MWCNTs enhanced the *FCR* of the S-UHPCs irrespective of the measurement method, as shown in Figure 15. FCR increased from 28.8 (M1S) to 39.2% (M2S) when the WSS measurement was used and increased from 25.2 to 34.5% when the DC measurement was used. The addition of copper wire meshes in the cube compressive specimens slightly decreased the compressive strength of specimens, because they may cause stress concentration. The ultimate strengths were 170.69 (*σ_p_* of M1S_WSS) and 156.07 (*σ_p_* of M2S_WSS) MPa for M1 and M2 specimens with copper wire meshes, respectively, as can be seen in Table 4, whereas they were 179 and 160 MPa for M1 and M2 specimens without copper wire meshes, respectively (Table 2).

The increase in compressive stress of the experimental cubes increased the strain, while the distance between adjacent conductive fillers decreased, and the number of connections of fillers increased. Thus, the contact and tunneling conduction of the composites increased, and the electrical resistivity of the composite consequently decreased [3,21]. Steel fibers affect contact conduction, whereas FSSAs affect tunneling conduction [3,21]. The addition of MWCNTs (nano-electrically conductive material) decreased the distance between FFs at the nano level and further enhanced tunneling conduction under compression. Thus, under compression, the S-UHPCs with MWCNTs resulted in a higher FCR compared to that of S-UHPCs without MWCNTs.

#### 4.2.3. Self-Damage Sensing of Smart UHPCs Using WSS under Tension

Similar to the DC measurement, the WSS measurement exhibited feasibility in measuring the electrical resistivity response under tension. The electrical resistivity of S-UHPCs significantly decreased when the tensile stress increased while generating multiple microcracks. The addition of MWCNTs increased the tensile strength and FCR of the S-UHPCs at the peak stress.

**Figure 16:**
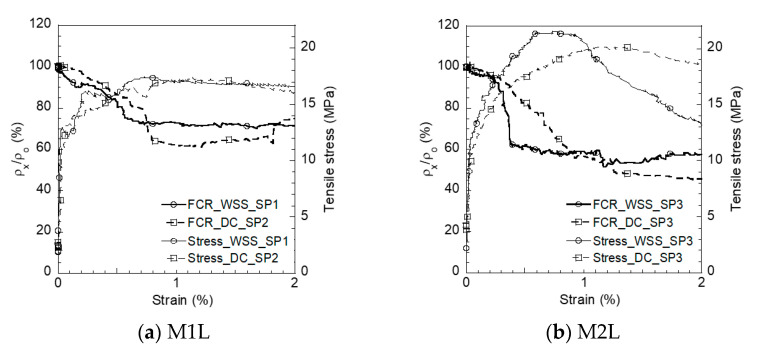
Electrical resistivity response of S-UHPCs under tension.

Figure 16 shows the electrical resistivity responses of the S-UHPCs using WSS and DC measurements under tension. Both measurement systems resulted in a similar electrical resistivity response to S-UHPCs, as reported in [25,26,27,28]. The tensile responses of S-UHPCs illustrated the strain-hardening behavior with the generation of multiple microcracks, which was attributed to a higher interfacial bond between the steel fiber bridging cracking and the UHPC matrix [29]. The electrical resistivity slightly decreased, given that the tensile stress was still within the elastic stage before the first cracking point. Subsequently, the electrical resistivity decreased when the high tensile stress increased until the post-cracking point, generating multiple microcracks. Thus, the damage to the S-UHPCs under tension can be easily detected based on the electrical resistivity response of S-UHPC measured by WSS measurements.

The fractional change in resistivity at both the first- and post-cracking points (*FCR_cc_* and *FCR_pc_*) was calculated to evaluate the strain sensing (elastic stage) and damage self-sensing after the matrix was subjected to cracking as follows:

(4) $$FCR_{cc}=\left|\frac{\rho_{cc}-\rho_{i}}{\rho_{i}}\right|\times 100=\left|\frac{\Delta \rho_{cc}}{\rho_{i}}\right|\times 100$$

(5) $$FCR_{pc}=\left|\frac{\rho_{pc}-\rho_{cc}}{\rho_{cc}}\right|\times 100=\left|\frac{\Delta \rho_{pc}}{\rho_{cc}}\right|\times 100$$

where *FCR_cc_* denotes the *FCR* at the first-cracking point, *FCR_pc_* denotes the *FCR* between the first- and post-cracking points, *ρ_cc_* denotes the electrical resistivity at the first-cracking point, *ρ_pc_* denotes the electrical resistivity at the post-cracking point, Δ*ρ_cc_* denotes the change in the electrical resistivity until the first cracking point, and Δ*ρ_pc_* denotes the change in the electrical resistivity from the first-cracking point to the post-cracking point.

To quantify the self-strain and damage sensing capacity of S-UHPCs, the strain and damage gauge factors (GFs) were calculated as follows:

(6) $$GF_{strain}=\left|\frac{FCR_{cc}}{\varepsilon_{cc}}\right|$$

(7) $$GF_{damage}=\left|\frac{FCR_{pc}}{\varepsilon_{pc}-\varepsilon_{cc}}\right|$$

where *GF_strain_* and *GF_damage_* denote the strain and damage GFs, respectively.

**Figure 17:**
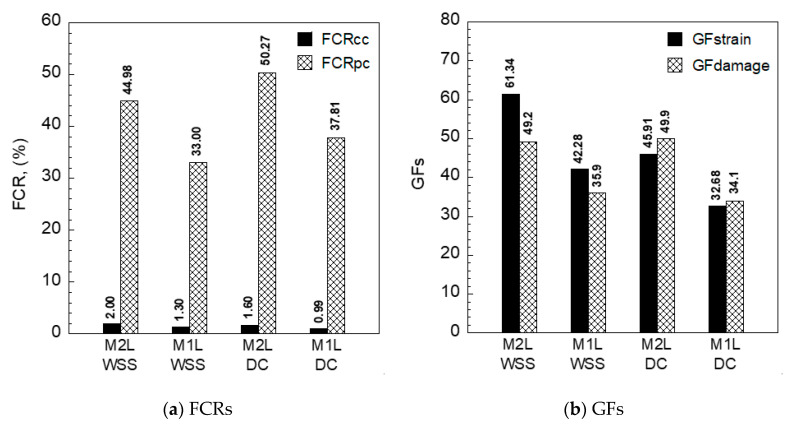
FCRs and GFs of S-UHPCs under tension.

Figure 17 compares the FCRs, *GF_strain_*, and *GF_damage_*, while Table 5 summarizes their values and the number of cracks under direct tension corresponding to different measurements. In the elastic stage, before the first cracking, the FCRs of S-UHPCs with MWCNTs (2.0 and 1.6% using WSS and DC measurements, respectively) slightly exceeded those of S-UHPCs without MWCNTs (1.30 and 0.99% using WSS and DC measurements, respectively). However, after matrix cracking, the FCRs of S-UHPCs with MWCNTs (44.97 and 50.97% using WSS and DC measurements, respectively) significantly exceeded those of S-UHPCs without MWCNTs (33.00% and 37.81% using WSS and DC measurements, respectively), irrespective of the measurement method. Furthermore, the FCR per crack of M2L (12.35% using WSS measurement) also exceeded that of M1L (9.36% using WSS measurement). In addition, the strain/damage GFs of S-UHPCs with MWCNTs (M2L) generally exceeded those of S-UHPCs without MWCNTs (M1L), as shown in Figure 17b.

The decrease in the electrical resistivities of S-UHPCs under tension, which resulted in the generation of multiple micro-cracks, was mainly dependent on the steel fiber bridging cracks and the number of multiple microcracks distributed to the bond between the steel fibers and UHPC matrix [30]. During the generation of matrix cracks, the electrical resistivity of the composite decreased, given that electrically conductive steel fibers bridged the crack [31,32]. Correspondingly, increases in the number of cracks increased the electrical resistivities of the S-UHPCs. The addition of MWCNTs enhanced the conductive network of S-UHPCs, i.e., the initial electrical resistivity of M2L (3023.67 kΩ-cm using the WSS measurement) was lower than that of M1L (4721.67 kΩ-cm using the WSS measurement). Therefore, given that the number of multiple cracks was slightly different (Table 5), the FCR calculated according to Equation (5) of M2L significantly exceeded that of M1L owing to its lower initial electrical resistivity. As shown in Equations (6) and (7), the GFs of M2L exceeded that of M1L because the tensile strains of M1L (*ε_cc_* = 0.032% and *ε_pc_* = 0.930% using WSS measurements; *ε_cc_* = 0.032% and *ε_pc_* = 1.110% using DC measurements) and M2L (*ε_cc_* = 0.034% and *ε_pc_* = 0.950% using WSS measurements; *ε_cc_* = 0.035% and *ε_pc_* = 1.016% using DC measurements) were also slightly different. The values of *GF_strain_* using WSS (61.34 and 42.28 for M2L and M1L, respectively) were always higher than those using DC measurement (45.91 and 32.68 for M2L and M1L, respectively), but those of *GF_damage_* using WSS (49.2 and 35.9 for M2L and M1L, respectively) and DC measurement (49.9 and 34.1 for M2L and M1L, respectively) were similar.

#### 4.2.4. Detecting Crack Position of S-UHPCs Using WSS with Multiple Measurements under Tension

**Figure 18:**
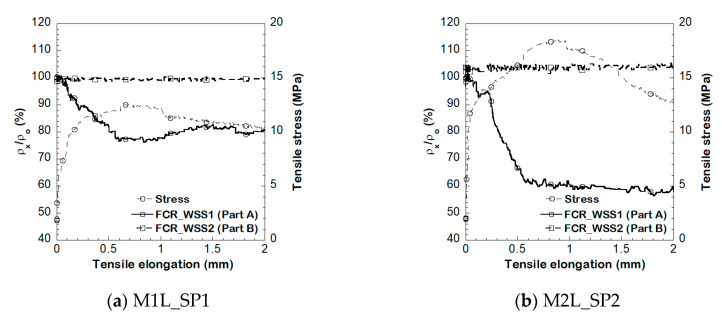
Electrical resistivity response of S-UHPCs using WSS with multiple channel measurements under tension.

To investigate the detecting crack position capacity of the S-UHPC under tension, we investigated the electrical resistivity response using WSS with multiple channel measurements. Figure 18 shows the electromechanical responses of different parts of the double-edged notch specimens by simultaneously using two WSSs. As shown in Figure 18, the electromechanical properties of S-UHPC at the part with a double-edged notch (Part A, WSS1) changed similar to that of the typical resistivity response of S-UHPCs. The part without the notch (Part B, WSS2) almost did not exhibit a change due to the generation of microcracks. A few cracks were generated under tension around the notch position in Part A owing to the decrease in the cross-sectional area of S-UHPC at the notched position. Conversely, cracks were not observed in Part B, without notches. Table 6 summarizes the FCR and the maximum stress (*σ_pc_*) formed in Part A. The FCR at Part A of M1L and M2L correspond to 30.6 and 37.6%, respectively. Based on the test results, the crack position in the S-UHPCs under tension can be detected based on the different electrical responses using WSS with multiple channel measurements.

## 5. Conclusions

This experimental study investigated the feasibility of WSS integrated with S-UHPCs for stress and damage sensing under compression and tension. Nano-sized FFs were favorable for enhancing the conductive network and sensing ability of the S-UHPCs. The main aspects of this study are as follows:

- A WSS was developed to measure electrical resistivity and broadcast measured response through a customized BLE beacon.
- The developed WSS has a power management circuit for long-term operation. With 10,000 mAh, the developed WSS can last 3.6 years without charging.
- The developed WSS is integrated with S-UHPCs to monitor the stress and damage of concrete composites under external loads to prove that it is a portable sensor with high accuracy, as a commercial DC wire measurement.
- Under compressive load, the WSS measurement measured higher electrical resistivity (14,086.18 and 12,411.52 kΩ-cm for M1 and M2, respectively) and *FCRs* (28.6% and 39.2% for M1 and M2, respectively) than those of DC meter measurement (9617.78 and 8570.34 kΩ-cm, 25.2% and 34.5% for the electrical resistivity and *FCR* of M1 and M2, respectively). The higher electrical resistivity of the S-UHPC using WSS rather than DC wire measurement is considered to be caused by a larger polarization effect due to a not fully optimized system code of WSS measurement.
- Under tension, the *FCRs* (37.81% and 50.27% for M1 and M2, respectively) using DC wire measurement were higher than those (33.00% and 44.98% for M1 and M2, respectively) using WSS measurement. On the other hand, the values of *GF_strain_* (42.28 and 61.34 for M1 and M2, respectively) using WSS measurement were larger than those (32.68 and 45.91 for M1 and M2, respectively) using DC wire measurement. The values of *GF_damage_* (35.9 and 49.2 for M1 and M2, respectively) using WSS measurement and those (34.1 and 49.9 for M1 and M2, respectively) using DC meter measurement showed similar values.
- A WSS with multiple measurement channels tested using a BLE beacon by defining a unique sensor ID for each WSS. The electrical resistivity responses at different positions were simultaneously obtained during loading. The crack position was easily indicated based on the electrical resistivity response.

The test results of the study proposed the development of WSS and its integration with S-UHPCs for monitoring stress and damage/cracks in smart structures under external loads. Multiple measurement channels can be easily used to detect the crack position of S-UHPC in actual smart structures. The developed S-UHPC is designed to embed in the structure, and to monitor the pressure change inside the structure through a developed wireless sensor.

## Figures and Tables

**Table 1:** Power consumption in Deep Sleep mode.

Device	Current Consumption (*I_IDLE_*)
DS3231 (RTC module)	110 μA
SN74LVC1G373 (D-type latch)	10 μA
74AUP1G00 (NAND gate)	0.9 μA
TPS22860 (Digital switch)	0.1 μA
MCP1725 (Voltage regulators)	120 μA
Electrical Resistance Sensing Circuit (S-UHPC: 1 MΩ + R1: 1 MΩ)	1.6 μA
**Total**	242.6 μA

**Table 2:** Composition by weight ratio, flow, compressive strength, and density of S-UHPCs.

No.	C	SF	SP	SS	FSSA	MWCNT	W	SPP	FS (vol%)	FL (vol%)	Flow (mm)	fc (MPa)	γ (kg/m^3^)
M1S	1	0.15	0.25	0.5	0.5	-	0.2	0.07	2	-	260	179	2423
M1L	1	0.15	0.25	0.5	0.5	-	0.2	0.07	-	2	260	179	2423
M2S	1	0.15	0.25	0.5	0.5	0.001	0.2	0.07	2	-	240	160	2573
M2L	1	0.15	0.25	0.5	0.5	0.001	0.2	0.07	-	2	240	160	2573

C: cement; SF: silica fume; SP: silica powder; SS: silica sand; W: water; SPP: superplasticizer; FS: short fiber; FL: long fiber; fc: compressive strength; γ: density.

**Table 3:** Properties of functional fillers.

Functional Filler	Size (mm)	Length (mm)	Density (g/cm^3^)	Elastic Modulus (GPa)
FSSA	<0.39	-	3.56	-
FS	0.2	6	7.85	200
FL	0.3	30	7.85	200
MWCNT	10^−5^	0.01	0.02–0.04	-

**Table 4:** Compressive–resistivity test results.

Notation	*σ_p_* (MPa)	*ρ_o_* (kΩ-cm)	*ρ_p_* (kΩ-cm)	Δ*ρ_p_* = *ρ_p_* − *ρ_o_* (kΩ-cm)	*FCR* = Δ*ρ_p_*/*ρ_o_* (%)
M1S_WSS					
Sp1	170.51	13,370.37	9787.04	3379.63	26.80
Sp2	170.86	14,157.41	9916.67	3898.14	29.95
Sp3	170.69	14,730.77	10,365.38	4250.00	29.63
Average	170.69	14,086.18	10,023.03	3842.59	28.80
STDV	0.14	557.66	247.80	357.49	1.42
M1S_DC					
Sp1	172.08	12,701.40	9337.33	3048.81	26.49
Sp2	184.33	11,958.98	8927.14	2744.00	25.35
Sp3	174.73	12,574.17	9582.15	2855.69	23.79
Average	177.05	12,411.52	9282.21	2882.83	25.21
STDV	5.26	324.18	270.23	125.91	1.11
M2S_WSS					
Sp1	153.53	9591.67	6033.3	3558.34	37.10
Sp2	149.03	8973.21	5250.0	3723.21	41.49
Sp3	165.66	10,288.46	6269.23	4019.23	39.07
Average	156.07	9617.78	5850.85	3766.93	39.22
STDV	7.02	537.27	435.64	190.68	1.80
M2S_DC					
Sp1	149.69	8340.47	5447.01	2620.71	34.69
Sp2	164.31	8851.31	6001.94	2499.85	32.19
Sp3	158.19	8519.25	5388.12	2960.74	36.75
Average	157.40	8570.34	5612.36	2693.77	34.55
STDV	5.99	211.66	276.52	195.12	1.86

STDV: standard deviation.

**Table 5:** Tensile resistivity test results.

Notation	*σ_pc_* (MPa)	*ρ_o_* (kΩ-cm)	Δ*ρ_pc_* (kΩ-cm)	*N_pc_*	*FCR_cc_* (%)	*FCR_pc_* (%)	*FCR/N_pc_* (%)	*GF_strain_*	*GF_damage_*
M1L_WSS									
SP1	17.45	5055	1380	3	1.48	27.30	9.10	54.95	37.4
SP2	17.96	4520	1345	3.5	0.88	29.76	8.50	22.69	30.9
SP3	19.45	4590	1925	4	1.53	41.94	10.49	49.20	38.16
Average	18.29	4721.67	1550	3.5	1.30	33.00	9.36	42.28	35.9
STDV	0.85	237.43	265.55	0.41	0.29	6.40	0.83	14.05	3.61
M1L_DC									
SP1	17.99	3959.49	1295.43	3	0.99	32.72	10.91	42.85	29.3
SP2	17.35	3505.23	1341.99	3	0.98	38.29	12.76	30.62	34.4
SP3	16.88	3444.99	1461.15	4	1.01	42.41	10.60	24.56	38.6
Average	17.41	3636.57	1366.19	3.33	0.99	37.81	11.42	32.68	34.1
STDV	0.45	229.66	69.79	0.47	0.01	3.97	0.96	7.61	3.80
M2L_WSS									
SP1	16.25	3081	1381	3.5	1.30	44.82	12.81	37.11	54.4
SP2	20.18	2860	1380	3.5	2.80	48.25	13.79	96.46	38.1
SP3	21.59	3130	1310	4	1.92	41.85	10.46	50.45	55.1
Average	19.34	3023.67	1357.0	3.67	2.00	44.98	12.35	61.34	49.2
STDV	2.26	117.45	33.24	0.24	0.61	2.62	1.39	25.42	7.85
M2L_DC									
SP1	20.27	2390.58	1393.42	4	2.17	58.29	14.57	65.64	61.6
SP2	17.66	2292.28	1090.78	3	1.74	47.58	15.86	40.36	46.1
SP3	20.20	2405.80	1081.10	3	0.89	44.94	14.98	31.77	42.1
Average	19.38	2362.89	1181.43	3.33	1.60	50.27	15.14	45.91	49.9
STDV	1.21	50.31	145.00	0.47	0.53	5.77	0.54	14.36	8.41

Δ*ρ_pc_* = *ρ_o_* − *ρ_pc_*; *N_pc_*: the number of cracks.

**Table 6:** Tensile resistivity test results of Part A in double-edged notch specimens.

Notation	*σ_pc_* (MPa)	*ρ_o_* (kΩ-cm)	*ρ_pc_* (kΩ-cm)	Δ*ρ* (kΩ-cm)	*FCR* (%)
M1L					
SP1	12.60	2995	2325	670	22.18
SP2	15.12	3260	1953	1307	40.11
SP3	16.53	2938	2068	870	29.62
Average	14.75	3064.33	2115.33	949	30.64
STDV	1.63	140.30	155.51	265.99	7.36
M2L					
SP1	18.53	1541.7	883.3	658.3	42.70
SP2	18.51	1550	925	625	40.32
SP3	17.14	1716.7	1208.3	508.3	29.61
Average	18.06	1602.8	1005.6	597.2	37.55
STDV	0.65	80.61	144.38	64.32	5.69
